# Edible Coatings from *Opuntia ficus-indica* Cladodes Alongside Chitosan on Quality and Antioxidants in Cherries during Storage

**DOI:** 10.3390/foods11050699

**Published:** 2022-02-26

**Authors:** Miltiadis V. Christopoulos, Dimitrios Gkatzos, Mina Kafkaletou, Jinhe Bai, Dimitrios Fanourakis, Giorgos Tsaniklidis, Eleni Tsantili

**Affiliations:** 1 Institute of Technology of Agricultural Product, Hellenic Agricultural Organization-DEMETER, S. Venizelou 1 Str., Lycovrissi, 14123 Attica, Greece; d.gkatzos@gmail.com; 2 Laboratory of Pomology, Department of Crop Science, Agricultural University of Athens, Iera Odos 75, 11855 Athens, Greece; mkafkaletou@gmail.com; 3 Horticultural Research Laboratory, USDA-ARS, 2001 S. Rock Rd., Ft Pierce, FL 34945, USA; jinhe.bai@usda.gov; 4 Laboratory of Quality and Safety of Agricultural Products, Landscape and Environment, Department of Agriculture, School of Agricultural Sciences, Hellenic Mediterranean University, Estavromenos, 71004 Heraklion, Greece; dimitrios.fanourakis82@gmail.com; 5 Institute of Olive Tree, Subtropical Plants and Viticulture, Hellenic Agricultural Organization, ELGO-Dimitra, P.O. Box 2228, 71003 Heraklion, Greece; giorgos.tsaniklidis@gmail.com

**Keywords:** *Prunus avium*, edible coatings, *Opuntia ficus-indica* extracts, chitosan, storage, quality, anthocyanins, phenolic compounds, total antioxidant capacity

## Abstract

The aim of this work was to investigate the effect of edible coatings (ECs) prepared from extracts of *Opuntia ficus-indica* (OFI) cladodes in comparison with a commercial chitosan formulation on the quality of ‘Regina’ cherries packaged in macro-perforated bags and stored for up to 28 d (1 °C, 90% RH). The coating concentrations were 25% and 50% aqueous OFI extract (approximately 0.59 and 1.18% dry matter, respectively), 1% OFI alcohol insoluble polysaccharide and 1% chitosan. The variables evaluated included weight loss (WL), respiration rates (RR), peel color, firmness, microbial decay, total antioxidants (phenolics, flavonoids, anthocyanins, antioxidant capacity), individual phenolic compounds (anthocyanins, hydroxycinnamic acids, flavan-3-*O*-ols), and pedicel removal force. The main results show that all coatings reduced WL and RR similarly, enhanced firmness throughout storage and antioxidants after 28 d of storage compared to the controls. Among treatments, chitosan resulted in much higher peel glossiness and firmness in comparison to OFI extracts. On day 28, all ECs resulted in higher antioxidants than controls, OFI extracts resulted in higher cyaniding-3-*O*-rutinoside than chitosan, while 50% OFI treatment resulted in the highest catechin concentration. Therefore, OFI extracts are promising ECs for cherry storage since they exhibited no negative effect, improved quality and extended storage life by one week compared to the controls.

## 1. Introduction

Cherries are early summer fruits and are highly appreciated by consumers due to their size, color and flavor. They are also a rich source of nutritive compounds, containing sugars, acids, potassium, melatonin, dietary fiber, vitamins C, A, E and B, phenolic acids and anthocyanins with only low caloric content [1]. Because of their composition, they contribute to health promotion and prevent diseases, such as cardiovascular disorders and types of cancer. Most of their beneficial effects are attributed to phenolic compounds [2].

Fresh cherries are exposed to the market for a very short period of time. They are very susceptible to physiological disorders and microbial decay, rendering the fresh produce sensitive to transport and storage. Moreover, they are harvested with pedicels, indicating the fruit’s freshness as long as they remain green and attached to the fruit [3]. Low-temperature management remains crucial for quality retention throughout the whole fresh cherry chain. Postharvest handling, such as ethanol treatment [4], modified clamshells with reduced water loss [5], or lowered oxygen concentration and elevated carbon dioxide in modified atmosphere packaging (MAP) in combination with low temperature maintain cherries’ sensory and quality characteristics for up to 6–7 weeks [6,7,8]. However, prolonging the shelf life of small and very sensitive fruit further still remains a challenge [9].

An alternative method, based on MAP, is the implementation of edible coatings (ECs). They form semipermeable barriers on the surface of each single fruit to moisture, solute and gas (O_2_, CO_2_ and other volatiles) transport, regulating their exchange between fruit and the surrounding atmosphere, provided that the fruit does not induce anaerobic respiration [10]. Therefore, the reduced water vapor pressure (WVP) caused by the coating results in reduced WL, O_2_ uptake and CO_2_ evolution and consequently reduced RR and in ripening delay. In stone fruit during storage, the effects of ECs composed of polysaccharides are the most studied, exhibiting reduced weight loss (WL) and ripening delay. They are applied to fruit as a liquid solution by immersion, spraying and dripping/brushing. Most studies use one of the two types of polysaccharide coating: chitosan or natural gel (mucilage) extracted from plant sources [10,11,12]. Chitosan is the most common edible coating applied to fruit, such as strawberries [13], sliced mangoes [14], and cherries [15,16,17], with promising effects on quality characteristics and storability [12] and inhibiting microbial decay [10,18].

Plant extracts applied to cherries, such as *Aloe vera* [19], guar gum with ginseng extract [20] and Arabic or almond gum [21], or to other fruit, such as *Aloe vera* to tomato [22] comprise a few examples exhibiting promising results during storage by delaying ripening processes. Recently, increasing interest has been focused on the novel ECs with material derived from wild plants that are rich in polysaccharides, such as the *Opuntia ficus-indica* (OFI) or *Opuntia cactus* (Cactaceae), commonly called prickly pear or cactus pear. It is a xerotrophyte plant, cultivated in Central and South America, Asia and South Europe. The mucilage of the cladodes has been studied in citrus [23], kiwi slices [24], strawberries [25], figs [26] and a few other cases, improving their shelf life, but not yet in cherries.

This work aimed to investigate some extracts of OFI cladodes (mucilage and gel solutions) alongside chitosan as ECs on cherry quality during low-temperature air storage. The variables evaluated were the fruit WL, respiration rates (RR), peel color, firmness, total phenolics (TP), total flavonoids (TF), total anthocyanins (TAN), total antioxidant capacity (TAC), individual phenolic compounds, pedicel removal force (PRF) and fruit microbial decay.

## 2. Materials and Methods

### 2.1. Plant Materials

Cherry (*P. avium* L.) fruits of cv. ‘Regina’ were harvested from trees grafted on ‘Gisela 6′ rootstock and grown in a commercial orchard located at Tegea, Arkadia County, Greece (37°26′ 20″ N, 22°24′47″ E). The fruit were harvested (16 July 2019) soon after the commercial maturity stage and transported to the laboratory within 2.5 h. Upon arrival at the laboratory, the fruits were sorted and only healthy fruits of uniform maturity, macroscopically free of disorders and diseases, were selected for the experiment. Cactus pear (*Opuntia ficus-indica*) cladodes were collected from vegetatively propagated/cladode rooting trees of a red/dark pink fruit variety grown in a commercial orchard located in Gerakas, Attica County, Greece (38°00′ 34″ N, 23°52′28″ E). One- and two-year old cladodes were collected on 1 July 2019, transported to the laboratory within 2 h and stored (4 °C, 85% RH) until processing (7–11 July 2019). 

### 2.2. Mucilage Extraction from Opuntia ficus-indica Cladodes and Preparation of the Edible Coatings

**Figure 1:**
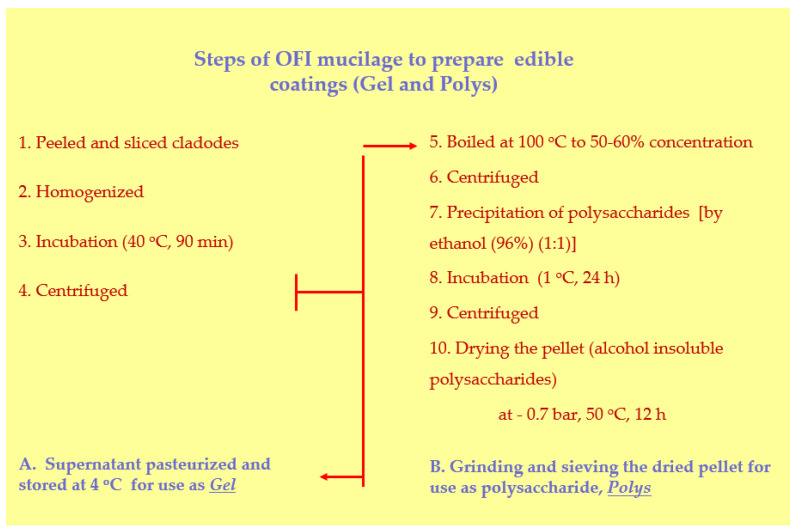
Diagram of the steps during preparation of edible coatings (ECs) from cladodes of *Opuntia ficus-indica* (OFI). A, the alcohol insoluble polysaccharides content, called Gel; B, the alcohol insoluble polysaccharides content, called Polys.

The mucilage from cladodes of cactus pear was extracted in two forms for the preparation of different ECs (Figure 1). The first mucilage material, aqueous polysaccharide extract, was prepared according to Del Valle et al. [25] with some modifications. Cladodes were peeled and sliced (1 cm width) (Step 1), and homogenized in a blender (Model CB15, Waring, Torrington, CT, USA) (Step 2). The slurry was incubated in a water bath at 40 °C for 90 min (Step 3) and then centrifuged (Model 4239R, Alc International, Srl, Cologno Monzese Srl, Cologno Monzese, Italy) at 10,000× *g* for 10 min (Step 4). The collected supernatant was thermally pasteurized in a water bath at 77 °C for 1 min and stored at 4 °C until use. The final material was in a gel (Code: Gel or G) form with specifications: 2.36% (*w/v*) dry matter; 0.69% (*w/v*) ash; 2.0 °brix; 4.47 pH. The second mucilage material was prepared according to Allegra et al. [24] with some modifications consisting of the alcohol-insoluble polysaccharide content of cladodes. This procedure was similar to the described aqueous-based polysaccharide extract, following the same steps 1–4, as a first part of the processing. The supernatant from step 4 was boiled at 100 °C until reaching a concentration of 50–60% of the initial volume (step 5), and then centrifuged at 3600× *g* for 5 min (Step 6). In the collected supernatant, an equal volume of ethanol (96%) was added (1:1) for precipitation of the polysaccharides (step 7) and the mixture was incubated at 1 °C for 24 h (step 8). After incubation, the mixture was centrifuged at 3600× *g* for 5 min (Step 9) and the pellet was collected containing the alcohol insoluble polysaccharides. Finally, the collected solid material was dried in a vacuum (−0.7 bar) oven at 50 °C for 12 h (Step 10), and the dried solid was ground and sieved (18 mesh) (Step 11) for use as the final cactus pear polysaccharide material (Code: Polys).

### 2.3. Edible Coating Treatments and Storage of Cherry Fruit

Preliminary experiments were conducted for the determination in the final edible coating application solution of the (i) concentration of the Gel material (tested 0, 20%, 25%, 33%, 50% and 100% Gel (*v/v*)); (ii) concentration of the Polys material (tested 0, 1%, 5% and 10% Polys (*w/v*)); (iii) concentration of plasticizer (tested 0%, 1%, 5% and 10% glycerol (*v/v*)); and (iv) an extra step for gelation (tested 0%, 1% and 5% CaCl_2_ (*w/v*)). Edible coating solutions were prepared (Gel ± glycerol, Polys ± glycerol) and applied by dipping treatments on cherry fruit followed or not by a successive gelation step (±CaCl_2_).

Increases in glycerol, CaCl_2_ and pH increase the viscosity but decrease the values of wettability and adhesion coefficients [12]. The selection criteria for the desired coating solution specifications and application procedure were the (i) uniform coverage of the whole fruit after drying of the coating; (ii) the presence of abnormal fruit appearance and (iii) fruit weight loss after 5 days at 1 °C, 85–90% RH. Based on the results of the preliminary experiments, 25% and 50% Gel and 1% Polys concentrations were selected, all with the addition of 5% glycerol as a plasticizer and no requirements for an extra gelation step.

For the final experiment, the tested coating solutions in deionized (DI) water were:

(i) a liter of 25% Gel (*v/v*) containing 5% glycerol (code: G25),
(ii) a liter of 50% Gel (*v/v*) containing 5% glycerol (code: G50),
(iii) a liter of 1% Polys (*w/v*) containing 5% glycerol (code: Polys),
(iv) a liter of 1% (*w/v*) chitosan in acetic acid (0.5% *v/v*) containing 5% glycerol,
(v) a liter DI water containing 5% glycerol used as a control for 7, 14, 21 and 28 d of storage.

The chitosan solution was prepared by dissolving 5 g of chitosan (Chitosan from shrimp shells; degree of deacetylation ≥0.75; color, white to beige; Aldrich Chemistry; Product) in 1 L of DI water containing 0.5% (*v/v*) acetic acid under stirring at 40 °C for 24 h.

Batches of about 300 g of cherries (a total of 6 batches per treatment) were dipped for 1 min in each coating solution, the excess coating was drained, and the coated fruits were dried under forced air at 20 °C for 60 min. Coated cherries were packaged in polypropylene (PP) macro perforated packages with 10 × 20 cm dimensions and 3 holes cm^−1^ perforation of 500 μm diameter (15 fruits per package, averaged 152.35 ± 10.98 g of fruit per package, 12 packages per treatment), sealed, and stored in a cold chamber at 1 °C and 90% RH for up to 28 days. Each package served as a biological replicate. Quality and chemical attributes were analyzed, just before coating treatments (day 0) and at 7, 14, 21 and 28 d after storage, on 3 replicates per treatment at each sampling day. Weight loss was measured immediately after removal from the store and packages, whereas the remaining variables were evaluated after removal from packages and temperature equilibration at 20 °C for 15 h. During each sampling, soon after the quality parameters’ evaluation on fresh fruit per package (10 per 15 fruit selected randomly), fruits were frozen (−20 °C) until the extraction of phytochemicals.

### 2.4. Total Soluble Solids, Titratable Acidity, pH, Peel Color, Weight Loss, Moisture/Dry Matter and Respiration

The total soluble solids (TSS) of the flesh was estimated in each fruit separately by an Atago 8469 (Atago Co., Ltd., Tokyo, Japan) hand refractometer. Titratable acidity (TA) was measured by the titration of 10 g of fruit sap to pH 8.2 with 0.1 M NaOH. pH was measured by a pH-meter (Jenway 3310; Jenway Ltd., Dunmow, UK).

The color grade was evaluated according to the color program developed by the Centre Technique Interprofessionnel des Fruit et Legumes (CTIFL, Paris, France), in which 1 = light pink and 7 = dark mahogany. The present cherries at harvest were evaluated as of color grade 6, indicating the advanced maturity stage.

Accurate peel color determinations were carried out on 10 fruits per replicate on the opposite sides of each fruit with a Minolta chromatometer (CR-300; Minolta, Ahrensburg, Germany) according to Tsantili et al. [27]. The measurements are expressed as chroma (intensity of color), hue angle (actual color, or redness), and L* value (lightness ranging from 0 = black to 100 = white). In particular, the recorded values of a* and b* were converted into hue angle (*h◦*) and C* according to the following equations:

*h◦* = tan^−1^ (b*/a*) when a* > 0 and b* > 0

*h◦* = 180^o^ + tan^−1^ (b*/a*) when a* < 0

*h◦* = 360^o^ + tan^−1^ (b*/a*) when a* > 0 and b* < 0

C* = (a* + b*)^1/2^

Fruit weight loss was measured immediately after removal of the packages from storage and expressed as the percentage difference between the fruit weight (15 cherries) immediately after drying at day 0 and the weight at sampling (%, *w/w*).

Fruit moisture/dry matter was determined according to AOAC method 934.06 on each sampling day by the difference in weight of ~5 g pulp from 10 fruit before and after drying at 105 °C until constant weight.

Fruit respiration rates (RR) assessed as CO_2_ production were measured using a closed portable infrared gas analyzer (LI-6400; LI-COR, Lincoln, NE, USA) connected to a 750 mL airtight jar at a flow rate of 900 μmol s^−1^ [28]. On each sampling day and for each coating treatment, the RRs were measured on 10 randomly selected fruits per replicate after temperature equilibration at 20 °C. The CO_2_ production rates were expressed in nmol kg^−1^ h^−1^.

### 2.5. Fruit Firmness and Microbial Decay

The texture analysis was performed using an HD-Plus texture analyzer (Stable Micro Pedicels Ltd., Godalming, UK) and the Texture Expert Exceed Software for the data analysis. The determination of the textural characteristics of whole fruits was conducted with a cylindrical probe of 2 mm diameter and movement speeds of 1 mm/s during the test, 5 mm/s for the pre-test and 10 mm/s for the post-test. The compression depth was set at 5 mm, the measurement was conducted at the equatorial zone in each fruit and the results were expressed as the maximum recorded force in N.

Each fruit was visually assessed for decay incidence (molds and other infections), and the presence (=1) or absence (=0) of any decay symptom was expressed as the mean of 10 fruit per replicate.

### 2.6. Resistance to Pedicel Removal

Resistance to pedicel removal (PRF) was measured with a HD-Plus texture analyzer equipped with a hook probe. Each fruit was immobilized in the moving probe, the tip of the pedicel was connected to the stable base of the instrument, and the probe was moving upwards in a perpendicular direction to the horizontal plane until pedicel removal. The movement speeds were 10 mm/s during the test, 10 mm/s for the pre-test, and 10 mm/s for the post-test, and the results were expressed as the maximum recorded force in N.

### 2.7. Extraction of Phytochemicals

The extraction procedure of phytochemicals was carried out according to Blackhall et al. [29] after some modifications. Frozen cherries (three replicates of 10 cherries each) were de-stoned and homogenized in a blender (Model 38BL40, Waring commercial, New Hartford, CT, USA) for 15 s. Approximately 2 g of cherry pulp and 20 mL of methanol containing 0.1% 10 N HCl were homogenized using an Ultra-Turrax (Model T25, Ika Labortechnik, Germany) for 1 min at 9500 rpm min^−1^. The homogenate was incubated in a supersonic bath for 60 min at 37 °C, centrifuged at 4000 rpm for 6 min and the supernatant was recovered and used for the analyses.

### 2.8. Determinations of Total Phenolics, Flavonoids, Anthocyanins and Antioxidant Capacity

Total phenolics (TP) was measured by the Folin–Ciocalteu method according to Tsantili et al. [30], recording the absorbance at 750 nm versus a blank using a spectrophotometer (Model Cary 50, Varian Inc., Walnut Creek, CA, USA). Total flavonoids (TF) were measured by a colorimetric method using a 0.3 mL cherry extract for reactions and absorbance recording at 510 nm [31], as described by Tsantili et al. [30]. Total anthocyanins (TAN) were measured according to Meyers et al. [31], as described by Tsantili et al. [30], recording absorbance at 510 and 700 nm in buffers at pH 1.0 and 4.5, and converted to cyanidin 3-rutinoside (keracyanin) equivalents (c-3-rut) using a molar extinction coefficient of 28,840 L mol^–1^ cm^–1^. Total antioxidant capacity (TAC) was evaluated using both ferric reducing antioxidant power (FRAP) [30] and radical scavenging capacity (2,2-diphenyl-1-picrylhydrazyl, DPPH) [32] assays according to Christopoulos and Tsantili [33]. For all determinations, triplicate reactions per replicate were performed, and the results of TP, TF, TAN and TAC were expressed as equivalents of gallic acid (GAE), catechin (CAE), c-3-rut and Trolox acid (TAE), respectively, all on a DW basis.

### 2.9. Determinations of Individual Phenolic and Anthocyanin Compounds

Individual phenolic and anthocyanin compounds were determined according to Durst and Wrolstad [34] by an HPLC system equipped with a pump Nexera X2 (LC-30 AD), an autosampler system (SIL-30AC), a diode array detector (SPDM20A) (Shimadzu, Kyoto, Japan) and a Macherey–Nagel HPLC column C18 (250 × 4.6; 5 μm, Nucleodur PolarTec at 30 °C. An aliquot of 5 mL of the extract of phytochemicals (point 2.7) was evaporated under N_2_ stream at 37 °C and the residue was dissolved in 1 mL MeOH (HPLC grade). The extract was filtered through a Chromafil AO-45/25 polyamide filter (0.45 μm pore size), 20 μL was injected and the flow rate was set at 1 mL min^−1^. The elution solvents were (A) 100% acetonitrile and (B) aqueous formic acid 1%. The separation of the compounds was achieved according to the gradient: 0–15 min, 35% A; 15–30 min, 10% A; 30–80 min, 15% A; 80–100 min, 50% A; and finally washing and reconditioning of the column (equilibration time), 100–105 min 5% A. Identification of compounds was carried out by comparing retention times and their UV–Vis spectra from 200 to 700 nm. Each compound was quantified in comparison with a multipoint calibration curve obtained from the corresponding authentic standard (Extrasynthese, Genay, France) and expressed as mg g^−1^ DW. Chlorogenic and neochlorogenic acid were monitored at 320 nm, flavan-3-ols at 280 nm and anthocyanins at 510 nm. The data analyses were carried out using LabSolutions LC/GC 5.82 (SkyCom, Tokyo, Japan).

### 2.10. Statistical Analyses

The significance of the treatment effects (Ecs), storage days and their interaction on the determined variables was estimated by two-way ANOVA. In controls, one-way ANOVA was also performed. During the last two sampling dates and also when denoted, partial analysis of data was performed in addition to two-way ANOVA of all data from day 7. Mean comparisons were performed using the Tukey-HSD multiple range test (α = 0.05) with standard error (SE) values calculated from the residual variances. Data of weight loss (WL), pedicel removal force (PRF), and the analyses of respiration rates (RR) of controls, hue angle of controls and pedicel removal force of controls were transformed to log10, while c-3-glc and analyses of controls of c-3-rut, catechin, epicatechin, chlorogenic and neochlorogenic acids were transformed to the square root. The data presented were back transformed. Decay data were analyzed without transformation after checking the residuals for normality, according to the Shapiro–Wilk test, and the plot of the residuals for homoscedasticity. All statistical analyses were performed using STATGRAPHICS Plus (Statgraphics Technologies Inc., The Plains, VA, USA).

## 3. Results

### 3.1. Total Soluble Solids, pH and Titratable Acidity

At harvest, the total soluble solids (TSS) was 18.33 ± 0.06 (%), pH 4.09 ± 0.06 and titratable acidity (TA) 0.49 ± 0.02% (*w/w*) malate (Table 1).

### 3.2. Fruit Color, Weight Loss and Respiration Rates

**Figure 2:**
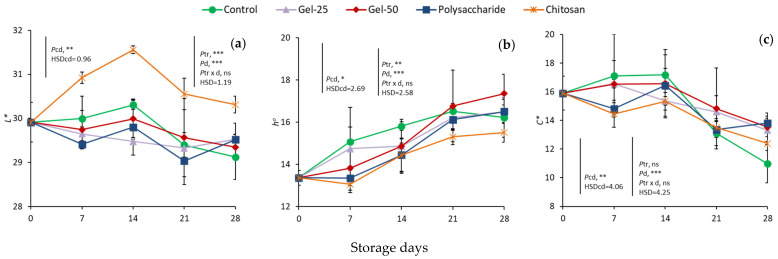
Effect of edible coatings on changes in peel color parameters, L* (**a**), hue angle (**b**) and C* (**c**), in cherries during storage. Points are means of three replicates of 10 cherries each; bars on the points, ± standard deviations. NS, non significant; * significant at *p* < 0.05; ** significant at *p* < 0.01; *** significant at *p* < 0.001. *Pcd*, probability of storage days in controls (one-way ANOVA from day 0); HSDcd, honest significant difference calculated from ANOVA. *Ptr*, probability of edible coating treatment; *Pd*, probability of storage days; *Ptr* *×* *d*, probability of interaction (two-way ANOVA from day 7); HSD, honest significant difference calculated from ANOVA.

The color parameter L* was 29.9 at harvest (Figure 2a). Changes in controls during storage were significant, exhibiting the highest value on day 14 and the lowest on day 28. Analysis of all data from 7 d showed that L* remained almost stable in controls, G25, G50 and Polys, whereas in chitosan-treated fruit, it increased substantially, reaching the highest value on day 14 and then decreased gradually, being significantly lower at the end of storage, but similar to the remaining treated fruit. The treatment effect and storage days were significant, but not their interaction.

Values of hue angle increased gradually during storage in controls from 13.4 on day 0 to 16.5 on day 21 and remained stable thereafter (Figure 2b). From day 7, increases in hue angle were observed in all samples, with chitosan-treated fruit exhibiting the lowest increases. The treatment effect and storage days were significant, but not their interaction.

In controls, the parameter C* was 15.9 at harvest and remained almost stable up to 14 d and then decreased until 28 d (Figure 2c). Factorial analysis showed the significant effect of days, but no effect of treatment or their interaction.

**Figure 3:**
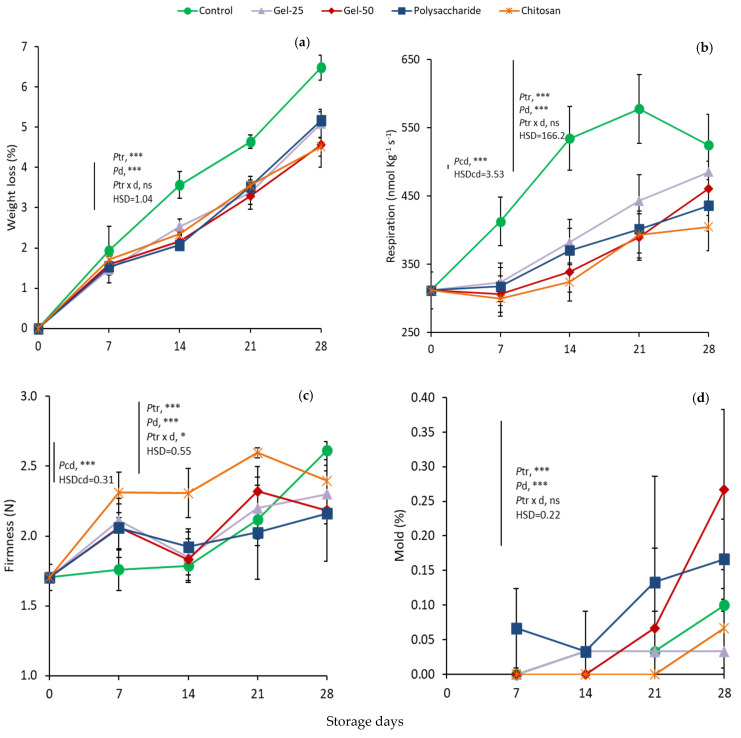
Effect of edible coatings on changes in weight loss (WL) (**a**), respiration (**b**), firmness (**c**) and microbial decay (**d**) in cherries during storage. Points are means of three replicates of 10 cherries each; bars on the points, ± standard deviations. NS, non significant; * significant at *p* < 0.05; *** significant at *p* < 0.001. *Pcd*, probability of storage days in controls (one-way ANOVA from day 0); HSDcd, honest significant difference calculated from ANOVA. *Ptr*, probability of edible coating treatment; *Pd*, probability of storage days; *Ptr* × *d*, probability of interaction (two-way ANOVA from day 7); HSD, honest significant difference calculated from ANOVA.

Weight loss (WL) averaged 1.61% on day 7 in all samples and increased progressively during storage up to day 28 (Figure 3a). WL in controls, being higher than in the remaining treatments from day 14 up to the end of storage, reached 6.48% after 28 d. WL increased in the other treatments, but at a slower and similar rate, averaging 4.86% on day 28. Indeed, the effect of treatments and days in storage were both highly significant, but not their interaction. The factor treatments, as the main effect, showed significant differences between controls and treatments, but no difference among treated fruit.

At harvest, CO_2_ production rates were approximately 311 nmol kg^−1^ s^−1^, but thereafter elevated sharply in controls up to 21 d, reaching 578 nmol CO_2_ kg^−1^ s^−1^, and then decreased (Figure 3b) (*Pcd* < 0.001). In all treated fruit, increases were consistent after 7 d, but much lower than controls throughout storage. The effect of treatment and storage days were both highly significant, in contrast to their non-significant interaction.

### 3.3. Fruit Firmness and Decay, and Pedicel Removal Force

The initial firmness value was 1.71 N, and after 14 d it increased gradually in controls, reaching the level of 2.61 N on day 28 (Figure 3c). However, chitosan-treated cherries showed the highest average levels in store (2.4 N).

In particular, chitosan exhibited a burst in firmness on day 7 (2.31 N), remained at almost stable levels up to day 14, peaked at 2.59 N on day 21 and declined to 2.39 N at the end of storage. Firmness in G25 and G50 also showed increased levels similar to the respective days in chitosan on days 7 and 21, while averaging 2.22 N between 7 and 28 d. Polys showed the lowest increases on average. The effect of treatments and days in storage were both highly significant, but not their interaction.

The mold development (%) of fruit increased during storage, with an overall mean of 0.05% and averaging 0.02% in both controls and chitosan and 0.026% in G25 during storage (Figure 3d). In Polys and G50, the decay averaged 0.084% and 0.1%, respectively. Additionally, on day 28, the decay increased in all treatments, being significantly higher in G50 than in G25.

**Figure 4:**
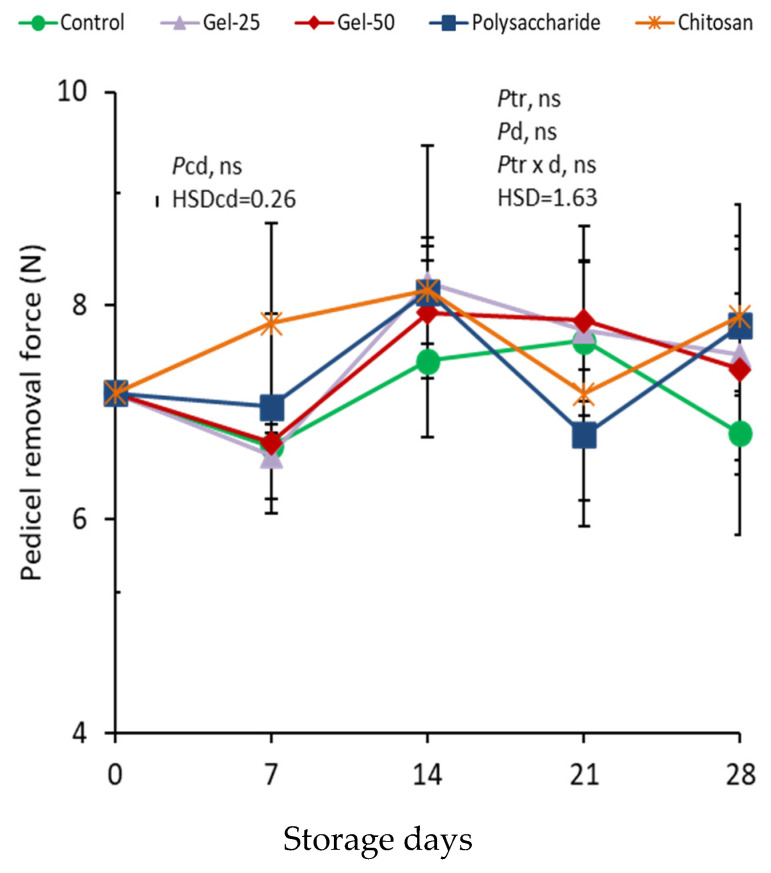
Effect of edible coatings on changes in pedicel removal force in cherries during storage. Points are means of three replicates of 10 cherries each; bars on the points, ± standard deviations. NS, non significant. *Pcd*, probability of storage days in controls (one-way ANOVA from day 0); HSDcd, honest significant difference calculated from ANOVA. *Ptr*, probability of edible coating treatment; *Pd*, probability of storage days; *Ptr* *×* *d*, probability of interaction (two-way ANOVA from day 7); HSD, honest significant difference calculated from ANOVA.

At harvest, pedicel removal force (PRF) in controls was 7.18 N and then fluctuated between 6.68 and 7.67 N (Figure 4). In all treatments, changes in PRF were not consistent during storage. The effects of treatments, storage days and their interaction were all insignificant.

### 3.4. Total Phenolics, Total Flavonoids, Total Anthocyanins and Total Antioxidant Capacity Determined with DPPH and FRAP Methods

**Figure 5:**
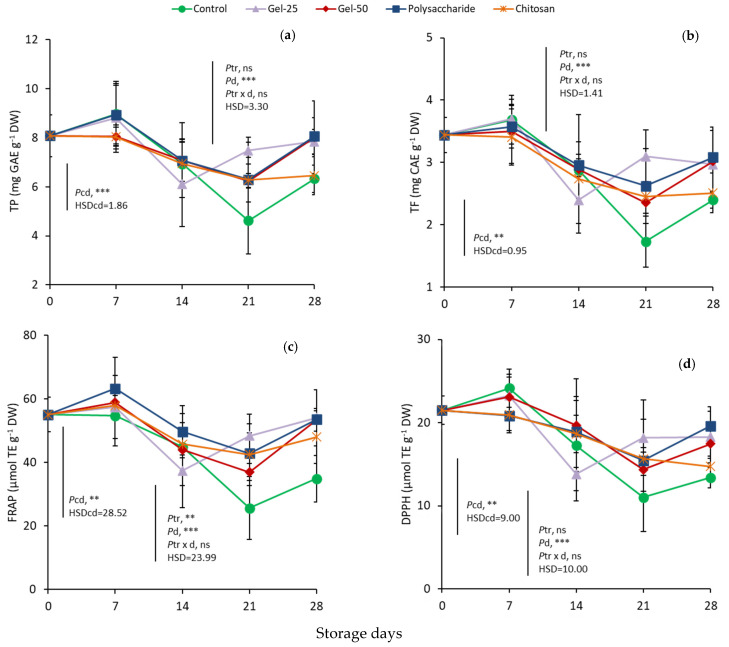
Effect of edible coatings on changes in total phenolic compounds (**a**), total flavonoids (**b**), total antioxidant capacity (TAC) determined by FRAP assay (**c**) and DPPH assay (**d**) in cherries during storage. Points are means of three replicates of 10 cherries each; bars on the points, ± standard deviations. NS, non significant; ** significant at *p* < 0.01; *** significant at *p* < 0.001. *Pcd*, probability of storage days in controls (one-way ANOVA from day 0); HSDcd, honest significant difference calculated from ANOVA. *Ptr*, probability of edible coating treatment; *Pd*, probability of storage days; *Ptr* *×* *d*, probability of interaction (two-way ANOVA from day 7); HSD, honest significant difference calculated from ANOVA.

At harvest, the values of TP, TF and TAN were 9.4 mg GAE g^−1^ DW, 3.3 mg CE g^−1^ DW and 4.5 mg c-3-rutg^−1^ DW (Figure 5a,b; Table 2). In controls, the values of all three variables decreased gradually and significantly up to day 21 and then increased to levels lower than at harvest. Treatments and controls showed a very similar pattern of changes during storage in each determined variable. The two-way analyses showed that treatments had no effect on changes in TP, TF and TAN in all three variables, but storage days were significant for TP and TF and the interaction of treatments with days was significant only for TAN. The lowest values of TP, TF and TAN were observed on day 21 in controls and reached approximately 4.6 mg GAE g^−1^ DW, 1.0 mg CE g^−1^ DW and 2.0 mg c-3-rut g^−1^ DW, respectively. In G25, the TP and TF values decreased on day 14 before the subsequent increases (partial analysis of data on day 14). Increases were observed in TP and TF variables in all treatments and controls on day 28, averaging 7.34 mg GAE g^−1^ DW, and 2.79 mg CE g^−1^ DW, respectively, compared to day 21, averaging 6.18 mg GAE g^−1^ DW, and 2.45 mg CE g^−1^ DW, respectively (partial analysis of data on days 21 and 28). The corresponding TAN values were 2.99 mg c-3-rut g^−1^ DW and 3.18 mg c-3-rut g^−1^ DW on days 21 and 28, respectively (partial analysis of data on days 21 and 28).

The patterns of changes in TAC values, determined with both methods, were similar to each other and close to the patterns observed in TP, TF and TAN. Initial samples showed 66.4 μmol TE g^−1^ DW with FRAP and 27.8 μmol TE g^−1^ DW with DPPH (Figure 5c,d). The lowest value in the controls was 25.7 μmol TE g^−1^ DW and 11 μmol TE g^- 1^ DW with FRAP and DPPH, respectively, and found on day 21 (*Pcd* < 0.01 for FRAP and DPPH). When all data from day 7 were analyzed, both treatment and storage days were significant for FRAP, but not their interaction (Figure 5c). In FRAP, controls averaged 40.06 μmol TE g^−1^ DW during storage, being the lowest average value among treatments. During storage, FRAP averaged 52.39, 49.33, 48.58, and 48.35 μmol TE g^−1^ DW in Polys, G25, chitosan and G50, respectively, with all these values being similar, but significantly higher than the respective values in controls. FRAP increased significantly to 48.82 μmol TE g^−1^ DW on day 28 on average, compared to 39.28 μmol TE g^−1^ DW on day 21. Additionally, FRAP decreased up to 14 d in G25 and increased thereafter, as in total antioxidants (TP, TF, TAN) and in contrast to the remaining treatments, which showed decreases up to day 21 and increases afterwards. On day 28, treated fruit exhibited similar FRAP values, but significantly higher than controls. However, DPPH values were not affected by treatment significantly, but only by days and the interaction of treatment with days, while changes followed those of FRAP (Figure 5d).

### 3.5. Anthocyanins

The major anthocyanin, cyanidin-3-*O*-rutinoside (c-3-rut), was 2.27 mg g^−1^ DW at harvest (Table 2). In the controls, it decreased gradually but significantly during storage, reaching the levels of approximately 1.13 mg g^−1^ DW on day 21, and remained almost stable thereafter. The two-way analysis from day 7 showed the significant effect of treatment and days, but not their interaction, with controls exhibiting the lowest values. Values of G25-treated fruit decreased from 7 d up to 14 d, reaching 1.15 mg c-3-rut g^−1^ DW, and then increased to 1.76 mg c-3-rut g^−1^ DW on day 21 and to 2.82 mg c-3-rut g^−1^ DW at the end of storage. C-3-rut in chitosan-treated fruit showed decreases after day 7, while it increased afterwards up to 1.86 mg c-3-rut g^−1^ DW on day 28. Partial analysis (data on 21 and 28 d) showed that cherries coated with G25, G50 and Polys averaged 2.95 mg g^−1^ DW, being 1.3-fold higher than the initial value, in contrast to the chitosan-coated and control fruits, being 0.82- and 0.66-fold lower, respectively.

Cyanidin-3-*O*-glucoside (c-3-glc) was found at 0.06 mg g^−1^ DW at harvest (Table 2), being at the highest level of the whole experiment, whereas the lowest level during storage was 0.01 mg g^−1^ DW on day 14 in the controls. In all treatments, c-3-glc decreased in the middle of storage, being lower in controls, G25 and chitosan than Polys and G50 on day 14, and then increased, averaging 0.2 mg g^−1^ DW in controls and chitosan and 0.5 mg g^- 1^ DW in the remaining treatments on day 28. The effect of days was significant in controls. The two-way analysis showed that the effect of treatment and days were significant, but not their interaction.

Peonodin-3-*O*-glucoside (p-3-glc) was 0.2 mg g^- 1^ DW at harvest (Table 2). In the controls, it decreased significantly to 0.02 mg g^−1^ DW on day 21 and showed an insignificant increase on day 28. When all data were analyzed, the treatment effect and storage days were also both significant, but not their interaction. During storage, c-3-glc averaged 0.06 mg g^−1^ DW in controls compared to the significantly higher values of the remaining treatments, averaging 0.13 mg g^−1^ DW, being approximately two-fold higher than the controls. On day 28, c-3-glc in all treatments also showed an increase, averaging 0.17 mg g^−1^ DW in comparison to the respective value of 0.07 mg g^−1^ DW on day 21. On day 28, the values in the controls were significantly lower than those in G25, G50 and Polys, which reached the levels at harvest, whereas those in chitosan were intermediate and close to those at harvest.

Malvidin-3-*O*-gluoside (m-3-glc) was 0.037 mg g^−1^ DW at harvest and remained almost stable in controls during storage. In the two-way analysis, the treatment effect and storage days were significant, but not their interaction. Values fluctuated from 0.02 to 0.072 mgg^−1^ DW, with the average values in the controls and G25 being 0.029 mg g^−1^ DW compared to 0.052 g^- 1^ DW in the other treatments. On day 28, in treated cherries, m-3-glc averaged 0.05 mg g^−1^ DW compared to 0.03 on day 21 mg g^−1^ DW, with controls and G25 being significantly lower than the remaining treatments on day 21.

### 3.6. Phenolic Acids and Flavan-3-ols

Chlorogenic acid was 0.02 mg g^−1^ DW initially and ranged from 0.01 to 0.05 mg g^−1^ DW, with the highest value in the controls observed on day 7 and the lowest on day 21 (Table 2), and the effect of storage days was significant. In the two-way analysis, the effect of storage days was significant, the effect of treatment was non-significant and that of the interaction was significant. In particular, chlorogenic acid averaged 0.19, 0.15, 0.14 and 0.17 mg g^−1^ DW on days 7, 14, 21 and 28, respectively, during storage.

Neochlorogenic acid, from 1.9 mg g^−1^ DW at harvest, decreased progressively during storage up to day 14 in G25, while up to day 21 in the remaining treatments (Table 2). The lowest value of neochlorogenic acid was observed in the controls on day 21, being 0.18 mg g^−1^ DW, but increased significantly thereafter (*Pcd* < 0.001). From the two-way analysis, the effect of storage days was significant, with the treatment effect and the interaction being non-significant. The averaged treatment values were 1.43, 1.02, 0.82 and 1.24 mg g^−1^ DW, on days 7, 14, 21 and 28, respectively.

In controls, catechin was 1.77 mg g^−1^ DW, decreased gradually during storage, reaching 0.61 mg g^−1^ DW on day 21 (Table 2), which was the lowest value in the whole experiment, and increased to 0.88 mg g^−1^ DW on day 28. Catechin in Polys- and G50-treated cherries exhibited significant changes during storage, but not consistent, averaging 2.37 and 2.69 mg g^−1^ DW, respectively, resulting in 1.85 and 2.15 mg catechin g^- 1^ DW, respectively, on day 28. In chitosan and G25, it decreased progressively during storage, reaching 1.2 and 1.41 mg g^−1^ DW at the end of storage. The effects of treatment, storage days and their interaction were all significant.

Epicatechin values were 0.22 mg g^−1^ DW at harvest (Table 2).The controls decreased until day 21 and increased at the end of storage significantly but slightly. When all data were analyzed, the treatments showed similar values which decreased up to day 21 and increased thereafter. The effect of treatment was not significant, but those of storage days and their interaction were significant. In all treated cherries, values averaged 0.20, 0.12, 0.09 and 0.21 mg g^−1^ DW on days 7, 14, 21 and 28, respectively.

Partial analysis of 21 and 28 d data (two-way ANOVA) showed the significant effect of treatment on chlorogenic, neochlorogenic, catechin and epicatechin, with the control values being the lowest in all cases, and all treatments equally effective in increasing the acids, whereas G50 was the most effective in increasing catechin.

## 4. Discussion

### 4.1. Peel Color

The present TSS, pH and TA values (Table 1) are in general agreement with other ‘Regina’ studies [35,36,37], and the present color values agree with those of the work of Harb et al. [6]. ‘Regina’ is appreciated by consumers who prefer the mahogany cherries, while at advanced maturity stage cherries obtain a good eating quality [38] with high antioxidant levels [39]. In this experiment, the control fruit deteriorated after 21 d of storage, in contrast to the treated ones that were marketable even after 28 d.

Chitosan treatment resulted in considerably higher L* values than all other cherries throughout storage, but in lower hue angle increases up to 21 d. Therefore, chitosan-treated cherries showed the best shininess with a mahogany color, exhibiting an appearance improvement [10]. Increases in hue angle during low-temperature storage were also found in ‘Regina’ controls harvested at a relatively high TSS and low hue angle values by others [6] and in alginate-treated cherries [40]. The increases in hue angle could be attributed to a loss of anthocyanins and/or to a lower rate of their synthesis. In other cherry studies, the hue angle decreased during storage [40], and the differences in hue angle changes could be attributed to the maturity stage at harvest. However, the decreases in hue angle were also reduced in treated ones with aloe [19], alginate [41], almond or Arabic gum [21] and chitosan [17,42]. In this work, because of the mahogany peel color, no browning could be observed.

### 4.2. Weight Loss and Respiration Rates

Here, the WL of controls, averaged 4.15% during storage, was comparable to other cherry studies [17,19,43,44]. Bai et al. [4] found that WL in macro-perforated packages was <1% after 6 weeks of storage at low temperature. Indeed, the perforated material and the number, area and frequency of perforation were different between the studies. In addition, the weight of fruit per package was very low in the present work, justifying the increased WL. Additionally, in this experiment there was no plan to calculate the contribution of the macro-perforated packages to the reduction in WL.

In ‘Regina’, the WL of the treated cherries was consistently and similarly reduced by all ECs in comparison to the controls from day 7 to the end of storage. Indicatively, the averaged WL of treated cherries was 0.74- and 0.75-fold lower than the controls on days 21 and 28, respectively. In cherries, it was shown that chitosan lowered WL [15,17], with the reduction being increased by increasing the chitosan concentration [15]. Similarly, reduced WL in cherries was achieved with other ECs [19,20,21] or in other species treated with OFI mucilage [24,25,26]. In another work on cherries, the increased hydrophobicity increased the reduction in WL and firmness [45]. However, WL results in lower fruit volume in cherries due to the lack of peel flexibility [46]. The incorporation of a plasticizer, such as glycerol, reduces the rigidity of the coating, by increasing its strength of elongation, although it also increases the WVP and WL. However, some cracks or flakes due to WL or mechanical damage are eliminated after the plasticizer’s addition [12]. Therefore, there is no recommendation for the hydrophobic/hydrophilic ratio. The properties of the coating depend on many factors, such the particular coating composition, conditions of storage and properties of the peel.

Cherries belong to non-climacteric fruit [43,47], although this classification is considered oversimplified [48]. Increases in ethylene production, although limited, and in RR were observed in ripening cherries [21,28], but these increases do not strictly comply with the non-climacteric behavior. Since the RR in cherries is considerable [7,43], and along with glycolysis reflects energy status, reduced RR is required to avoiding consuming high energy levels [6]. Reduced RR increases were observed in cherries treated with guar gum [20], almond or Arabic gum [21], chitosan [15], as well as in strawberries with OFI mucilage [25]. On the contrary, in several cherry studies, the RR decreased during storage, but ECs again exhibited their beneficial effect on increased reductions [17]. The difference in the direction of respiration changes is attributed to the maturity stage at harvest and conditions during and after storage.

Here, the rates of respiration are comparable to other cultivars [20,28], while all ECs exhibited a similarly positive effect on reduced RR. On day 21, the RR of G25-, G50-, Polys- and chitosan-treated fruit was lower than controls by 0.77-, 0.66- 0.7- and 0.68-fold, respectively, while on day 28 it was lower by 0.92-, 088-, 0.83- and 0.77-fold.

### 4.3. Firmness

The final firmness values comprise the result of non-equivalent rates of softening and of increases in firmness due to WL [28]. A similar trend of firmness increases in cherries during storage was found in three out of six cultivars [49] and in alginate-treated ones [40]. This discrepancy between the increased and decreased firmness is associated with the cultivar [49], maturity stage and the conditions during their shelf life.

In this work, the contribution of all ECs to firmness increases seems to be higher than that of WL, as compared to the firmness and WL of controls (Figure 3a,c). These results are in line with chitosan or other ECs applied to cherries [19,20,21,41,50] or to other commodities [12], and with OFI mucilage on strawberries [25] and cut kiwi fruit [24]. However, here, the best effect on firmer fruit was observed by chitosan throughout storage, by comparison.

The present beneficial effect of treatments on firmness could be ascribed, at least partially, to the lower enzyme activities related to the firmness, and has been demonstrated by the enzyme activities and their reaction products. The chitosan effect on reduced softening in Chinese cherries was explained by the reduced gene expression of pectin methylesterase (PME) genes, PME activity, the lower content of sodium carbonate soluble pectins (SCSP), the lower rate of pectin demethylation and the loss of main and side chain neutral sugars of rhamnogalacturonan I (primary structure of SCSP in cherries) [50]. Additionally, in kiwi slices, the higher firmness of the tissue treated with OFI mucilage was attributed to the higher total pectin and protopectin concentrations during storage, implying the lower respective enzyme activities in comparison to controls [24]. Enzyme activities connected with firmness loss require O_2_ and ethylene. Here, the levels of O_2_ were reduced by ECs, but it was not known whether chitosan that resulted in higher firmness suppressed the O_2_ levels more than the other ECs used. Ethylene synthesis is low in cherries, but there is no ethylene limit that inactivates ethylene action even under low temperatures. Moreover, increases in ethylene along with firmness loss and enhancement of SP content have been observed in cherries during low-temperature air storage [28].

### 4.4. Microbial Decay, Pedicel Removal

Ripe fruits are very vulnerable to microbial decay. Chitosan is known to prevent the fungal decay of fresh produce. Its positive charge of amino groups reacts with the negatively charged microbial membrane, inhibiting DNA replication, but also it binds to metals, inhibiting microbial growth [18]. Additionally, it triggers fruit defense responses by increasing the activities of chitinase, β-1,3 glucanase (directly preventing the microbial growth) and phenylalanine ammonia lyase (PAL) (inducing the synthesis of phenolic compounds) [18]. Here, although G50 and Polys exhibited higher decay than the other samples on day 28, the decay still remained at very low levels. Moreover, decay was also limited in controls during storage. Although glycerol has antimicrobial activity [51], it is suggested that decay prevention was the main outcome from the small fruit groups. Allegra et al. [24] found that OFI mucilage increased the growth of yeast slightly in kiwi slices, whereas it resulted in the low development of *Enterobacteriacea* in figs [26]. Further research is needed to investigate the effect of these ECs on the decay of cherries packaged in larger groups.

Pedicel removal force (PRF) is an indicator of the adhesion and retention of pedicels during postharvest life, which is essential to reduce WL and microbial contamination. Additionally, the appearance of the attached fresh green pedicels plays a crucial role in market value. Here, the PRF was not affected by treatment, while it exhibited inconsistent changes during storage, which might be the interaction of ECs, and WL of both fruit and pedicels. In other studies of uncoated cherries, PRF decreased [35,43] in contrast to this work.

### 4.5. Total Antioxidants and Total Antioxidant Capacity

Mahogany cherries exhibit higher antioxidant concentrations than those with a bright red color [38]. The present results comply well with other studies on ‘Regina’ [36,52] and are rather close to the highest values among cherry cultivars [35,53].

Here, it is of interest that the patterns of changes in TP, TF and TAN during storage had very close similarity, exhibiting a decline up to day 21 d. It is known that cherry antioxidants increase during ripening [39], and it seems that maxima concentrations of TP, TF and TAN in the ripe ‘Regina’ were observed at harvest. However, an increase was observed in total antioxidants on day 28 at levels up to the initial ones. All ECs and controls resulted in similar decreases and increases during storage.

There has been an argument about the effect of low temperature storage on cherry anthocyanin changes. For example, the elevation of anthocyanin concentration was found by Gonçalvez et al. [54,55], in contrast to decreases presented by others [17]. A further study on cherries with regard to this issue found reduced transcription of genes coding for the enzymes anthocyanidin synthase (ANS) and flavonol 3-*O*-glucosyltransferase (UFGT) (crucial enzymes for anthocyanin synthesis) and of PAL (the initial enzyme of the phenylpropanoid pathway) and limited TAN increase, but stable TP levels in cherries at low non-chilling temperature compared to those at harvest, indicating a complex regulation of phenolic compounds [56]. Here, it is suggested that ripe cherries had probably almost exhausted their ability to synthesize more phenolic compounds, at least at low temperature, whereas an effort to recover was observed towards the end of storage. The decreases might be the result of the deceleration phenolic synthesis under low temperature [56] and their depletion to defend the reactive oxygen species (ROS) that were inevitably produced after harvest [57]. Indeed, a pattern of PAL activity shown by Dang et al. [15] during air storage complies well with the present pattern of TP, TF and TAN. In addition, in this work TAN decreases are in general agreement with hue angle increases.

Chitosan treatment in other cherry studies resulted in lower decreases in antioxidants than controls [17]. Enhanced activities of PAL and antioxidant enzymes and prevention of PPO and lipoxygenases (LOX) due to chitosan treatment contributed to the extension of cherry storage life [15,16]. PAL increases at late periods of storage were also attributed, at least partially, to chitosan effect [18], and all these comply with most antioxidant changes here. Nevertheless, the effectiveness of any EC on fruit depends on the cultivar and/or its composition/properties as well as the method of preparation and application. For example, increasing the alginate concentrations above 1%, as an edible coating in one study [40], but also decreasing to 1% in another study [41], had an impact on extending storage life, maintaining antioxidants at elevated levels in stored cherries. Additionally, guar gum applied to cherries maintained a higher concentration of ascorbic acid and increased TP levels, but suppressed the TAN levels during cold storage compared to controls [20], indicating the complexity of phenolic compound synthesis as well. OFI mucilage on figs in the store did not have any effect on TP, but a positive one on total carotenoids [26].

Generally, TAC renders the cherries as a source of natural antioxidants [2]. Anthocyanins comprise a large part of the TP amount, with c-3-rut primarily contributing to TAN [52]. C-3-rut possesses a high antioxidant capacity [58]. ‘Regina’, being a dark-colored cultivar, exhibited a relatively high TAC determined either by the FRAP or DPPH assays, similar to another ‘Regina’ study [37]. The different results obtained between the FRAP and DPPH assays here were expected and agree with another cherry study [43], since they are based on different methods. It is important that all EC-treated cherries possessed similarly higher FRAP levels (Figure 5c) than controls after 21 and 28 d of storage.

Considering that controls were non-marketable after 28 d of storage due to their appearance (attributed to high WL), the present results confirm the beneficial effect of coatings not only on extending the cherry storage, but also on maintaining the antioxidant concentrations close to the initial levels.

### 4.6. Phenolic Compounds

Phenolic compounds also play an important role in cherry quality since they influence the appearance, taste and nutritional value of fruit [1]. The most representative classes of cherry phenolic compounds are anthocyanins, hydroxycinnamic acid derivatives and flavan-3-ols, while flavanols (such as rutin) have also been determined [1,54,59].

C-3-rut is the major anthocyanin in cherries, comprising approximately up to 95% of TAN [1]. The present values of c-3-rut ranging between 1.13 and 3.07 mg g^−1^ DW, are in agreement with other cherry studies [36,52]. It is of interest that the averaged c-3-rut values in controls were the lowest throughout storage, whereas those in fruit coated with OFI extracts were the highest after 28 d of storage, and indeed were higher than the initial values. P-3-glc showed a similar trend of changes, but after 28 d of storage the levels of all treated cherries with ECS were close to the initials, being 2.4-fold higher than controls. C-3-glc and m-3-glc were minor anthocyanins in ‘Regina’. The present c-3-glc values are very similar to other ‘Regina’ studies [36,52], while p-3-glc and m-3-glc are in accordance with those found in other cultivars by Gonçalvez et al. [54] and Martini et al. [59], respectively. Regarding the acids chlorogenic and neochlorogenic, as well as the flavan-3-*O*-ols catechin and epicatechin, their concentrations are comparable with other ‘Regina’ studies [36,52], while their changes followed the pattern of anthocyanins. At the end of storage, G50 exhibited the highest catechin concentration.

Therefore, at the end of storage, the OFI extracts were the most effective in containing higher c-3-rut levels even than the initial ones, while G50 in the highest catechin concentration.

## 5. Conclusions

The results here show that all ECs reduced WL and RR similarly and increased firmness values compared to the controls, resulting in extending the cherry storage by one week. Among treatments, chitosan was superior in enhancing peel glossiness and firmness when compared to OFI extracts. OFI extracts exhibited elevated c-3-rut and G50 catechin levels after 28 d of storage and no negative effect among the determined variables.

The use of raw materials from plant residues is desirable, while OFI cladodes are rich in dietary fibers, calcium, potassium, carbohydrates and polyphenols. Nevertheless, *Opuntia* genotype, cladode age, cultivation area and pruning season affect their composition. Cladodes are abundant after pruning in cultivation areas of cactus pear, and comprise an alternative for ECs preparation at low cost, while in a dried form would facilitate their storage and transport [60]. In OFI cladode extracts, incorporation of other materials or compounds along with plasticizers could improve the properties and functionalities of the coating. However, more experiments are needed to improve the effectiveness of OFI extracts on cherry storage. After all, *Opuntia* cladode extracts have potential as ECs to extend the storage life and improve the quality of cherries.

## Figures and Tables

**Table 1:** Total soluble solids (TSS), pH values and titratable acidity (TA) of juice in cherries at harvest.

Harvest		
TSS ^a^ (%)	pH	TA (%, *w*/*w*)
8.33 ± 0.06	4.09 ± 0.06	4.29 ± 0.02

^a^ Numbers are means of three replicates of 10 cherries each ± standard deviations.

**Table 2:** Effect of edible coatings on changes in total antioxidant anthocyanin content, on cyaniding-3-*O*-rutinoside, cyaniding-3-*O*-glucoside, peonidin-3-*O*-glucoside, malvidin-3-*O*-glucoside, chlorogenic acid, neochlorogenic acid, catechin and epicatechin in cherries during storage.

		Storage Days										
	Treatments	0	7	14	21	28	*P* *cd*	HSDcd	*P* *tr*	*P* *st*	*Ptr* × *st*	HSD
TAN (mg g^−1^ DW)	Control	4.56 ± 1.13	3.56 ± 0.83 ^a^	3.02 ± 0.86	2.04 ± 0.83	2.90 ± 0.19	ns ^b^	2.72 ^c^	ns	ns	**	1.93 ^d^
	Gel-25	4.56 ± 1.13	3.30 ± 0.38	2.25 ± 0.90	3.45 ± 0.74	3.56 ± 0.53						
	Gel-50	4.56 ± 1.13	3.53 ± 0.51	2.34 ± 0.82	2.51 ± 0.30	3.64 ± 0.54						
	Polysaccharide	4.56 ± 1.13	4.21 ± 1.12	2.83 ± 0.94	2.70 ± 0.30	4.00 ± 0.16						
	Chitosan	4.56 ± 1.13	3.10 ± 0.73	2.41 ± 0.43	2.50 ± 0.52	2.95 ± 0.26						
Anthocyanins												
Cyanidin 3-*O*-rutinoside (mg g^−1^ DW)	Control	2.27 ± 0.53	1.75 ± 0.41	1.28 ± 0.34	1.13 ± 0.38	1.51 ± 0.15	*	1.35	***	***	ns	1.37
	Gel-25	2.27 ± 0.53	1.80 ± 0.20	1.15 ± 0.31	1.76 ± 0.18	2.82 ± 0.41						
	Gel-50	2.27 ± 0.53	2.37 ± 0.22	1.83 ± 0.60	1.73 ± 0.21	3.07 ± 0.41						
	Polysaccharide	2.27 ± 0.53	2.27 ± 0.26	1.95 ± 0.87	1.65 ± 0.19	2.95 ± 0.69						
	Chitosan	2.27 ± 0.53	2.26 ± 0.08	1.35 ± 0.23	1.43 ± 0.24	1.86 ± 0.14						
Cyanidin 3-*O*-glucoside (mg g^−1^ DW)	Control	0.056 ± 0.006	0.025 ± 0.022	0.005 ± 0.003	0.016 ± 0.014	0.023 ± 0.005	**	0.01	*	***	ns	0.001
	Gel-25	0.056 ± 0.006	0.023 ± 0.011	0.004 ± 0.004	0.020 ± 0.004	0.051 ± 0.016						
	Gel-50	0.056 ± 0.006	0.024 ± 0.003	0.024 ± 0.008	0.019 ± 0.002	0.052 ± 0.016						
	Polysaccharide	0.056 ± 0.006	0.031 ± 0.008	0.022 ± 0.020	0.019 ± 0.004	0.046 ± 0.020						
	Chitosan	0.056 ± 0.006	0.028 ± 0.007	0.007 ± 0.006	0.011 ± 0.010	0.024 ± 0.009						
Peonidin 3-*O*-glucoside (mg g^−1^ DW)	Control	0.104 ± 0.017	0.062 ± 0.031	0.063 ± 0.022	0.020 ± 0.017	0.082 ±0.007	**	0.05	***	***	ns	0.11
	Gel-25	0.104 ± 0.017	0.135 ± 0.037	0.072 ± 0.052	0.076 ± 0.040	0.192 ± 0.069						
	Gel-50	0.104 ± 0.017	0.158 ± 0.023	0.133 ± 0.013	0.097 ± 0.020	0.208 ± 0.017						
	Polysaccharide	0.104 ± 0.017	0.185 ± 0.007	0.106 ± 0.021	0.078 ± 0.026	0.201 ± 0.025						
	Chitosan	0.104 ± 0.017	0.167 ± 0.035	0.110 ± 0.011	0.085 ± 0.034	0.169 ± 0.025						
Malvidin 3-*O*-glucoside (mg g^−1^ DW)	Control	0.037 ± 0.009	0.035 ± 0.014	0.025 ± 0.004	0.029 ± 0.016	0.030 ± 0.004	*	0.01	***	**	ns	0.03
	Gel-25	0.037 ± 0.009	0.017 ± 0.001	0.026 ± 0.011	0.029 ± 0.007	0.042 ± 0.026						
	Gel-50	0.037 ± 0.009	0.051 ± 0.007	0.051 ± 0.006	0.044 ± 0.009	0.067 ± 0.006						
	Polysaccharide	0.037 ± 0.009	0.072 ± 0.008	0.049 ± 0.008	0.041 ± 0.006	0.065 ± 0.009						
	Chitosan	0.037 ± 0.009	0.050 ± 0.011	0.048 ± 0.012	0.039 ± 0.014	0.054 ± 0.003						
Phenolic acids												
Chlorogenic acid (mg g^−1^ DW)	Control	0.023 ± 0.010	0.043 ± 0.018	0.022 ± 0.004	0.012 ± 0.002	0.019 ± 0.001	*	0.001	ns	***	*	0.01
	Gel-25	0.023 ± 0.010	0.029 ± 0.001	0.019 ± 0.005	0.021 ± 0.003	0.033 ± 0.003						
	Gel-50	0.023 ± 0.010	0.041 ± 0.005	0.020 ± 0.016	0.024 ± 0.002	0.036 ± 0.004						
	Polysaccharide	0.023 ± 0.010	0.039 ± 0.003	0.033 ± 0.006	0.022 ± 0.004	0.036 ± 0.002						
	Chitosan	0.023 ± 0.010	0.035 ± 0.005	0.032 ± 0.004	0.028 ± 0.006	0.031 ± 0.002						
Neochlorogenic acid (mg g^−1^ DW)	Control	0.62 ± 0.03	0.43 ± 0.06	0.37 ± 0.09	0.18 ± 0.04	0.35 ± 0.01	***	0.01	ns	***	ns	0.61
	Gel-25	0.62 ± 0.03	0.49 ± 0.03	0.29 ± 0.12	0.37 ± 0.06	0.58 ± 0.09						
	Gel-50	0.62 ± 0.03	0.74 ± 0.12	0.49 ± 0.19	0.37 ± 0.03	0.63 ± 0.04						
	Polysaccharide	0.62 ± 0.03	0.70 ± 0.06	0.50 ± 0.13	0.32 ± 0.04	0.60 ± 0.06						
	Chitosan	0.62 ± 0.03	0.61 ± 0.06	0.46 ± 0.05	0.44 ± 0.09	0.53 ± 0.04						
Flavan-3-ols												
Catechin (mg g^−1^ DW)	Control	1.77 ± 0.16	1.07 ± 0.05	1.08 ± 0.23	0.61 ± 0.08	0.88 ± 0.07	**	0.01	***	**	ns	1.02
	Gel-25	1.77 ± 0.16	1.27 ± 0.17	1.03 ± 0.20	1.13 ± 0.09	1.41 ± 0.13						
	Gel-50	1.77 ± 0.16	1.55 ± 0.17	1.98 ± 0.71	1.54 ± 0.37	2.15 ± 0.27						
	Polysaccharide	1.77 ± 0.16	1.45 ± 0.09	2.02 ± 0.67	1.00 ± 0.06	1.85 ± 0.42						
	Chitosan	1.77 ± 0.16	1.66 ± 0.49	1.46 ± 0.19	1.21 ± 0.17	1.20 ± 0.11						
Epicatechin (mg g^−1^ DW)	Control	0.22 ± 0.06	0.20 ± 0.14	0.06 ± 0.01	0.04 ± 0.01	0.07 ± 0.01	***	0.01	ns	***	**	0.05
	Gel-25	0.22 ± 0.06	0.11 ± 0.03	0.07 ± 0.04	0.06 ± 0.04	0.19 ± 0.04						
	Gel-50	0.22 ± 0.06	0.15 ± 0.03	0.14 ± 0.02	0.11 ± 0.01	0.23 ± 0.02						
	Polysaccharide	0.22 ± 0.06	0.18 ± 0.01	0.11 ± 0.03	0.09 ± 0.03	0.20 ± 0.03						
	Chitosan	0.22 ± 0.06	0.18 ± 0.02	0.12 ± 0.01	0.10 ± 0.03	0.17 ± 0.02						

^a^ Numbers are means of three replicates of 10 cherries each ± standard deviations. ^b^ NS, non significant; * significant at *p* < 0.05; ** significant at *p* < 0.01; *** significant at *p* < 0.001. ^c^ *Pcd*, probability of storage days in controls (one-way ANOVA from day 0); HSDcd, honest significant difference calculated from ANOVA. ^d^ *Ptr*, probability of edible coating treatment; *Pd*, probability of storage days; *Ptr* *× d*, probability of interaction (two-way ANOVA from day 7); HSD, honest significant difference calculated from ANOVA.

## Data Availability

Data is contained within the article.
